# Local Linear Wavelet Neural Network–Based Unscented Kalman Filter for Vehicle Collision Estimate Warning System and Ensuring Stable Vehicle-to-Infrastructure Communication

**DOI:** 10.1155/abb/2451501

**Published:** 2024-12-22

**Authors:** Yonas Kebede Lema, Satyasis Mishra, Demissie J. Gelmecha

**Affiliations:** ^1^Department of ECE, Adama Science and Technology University, Adama, Ethiopia; ^2^Department of ECE, Centurion University of Technology and Management, Bhubaneswar, Odisha, India

**Keywords:** advanced driver assistance systems, driver inattention, Kalman filter, local linear wavelet neural network, unscented Kalman filter

## Abstract

The accident mortality rates are rapidly increasing due to driver inattention, and traffic accidents become a significant problem on a global scale. For this reason, advanced driver assistance systems (ADASs) are essential to enhance traffic safety measures. However, adverse environmental factors, weather, and light radiation affect the sensors' accuracy. Furthermore, potential risks may go unreported if they obstruct the sensor's line of sight or are outside its limited field of view. To overcome these problems, this research presents a vehicle collision estimate warning system that leverages a combined approach of a local linear wavelet neural network (LLWNN) and an unscented Kalman filter (UKF). The system integrates sensor data and vehicle-to-everything (V2X) communication to enhance the accuracy and reliability of vehicle state estimation and collision prediction. The LLWNN module is responsible for forecasting the future states of the vehicle based on historical sensor measurements. This powerful time-series modeling technique allows the system to anticipate the vehicle's trajectory and potential collision risks. The UKF then optimally fuses the LLWNN predictions with the real-time sensor data, including information received through V2X communication, to generate accurate, up-to-date estimates of the vehicle's state. The V2X technology enables the seamless exchange of critical safety information between the host vehicle, surrounding vehicles, infrastructure, and other road users. This includes data on vehicle position, speed, acceleration, and intended maneuvers. By incorporating this V2X-based situational awareness, the system can better perceive the dynamic traffic environment and identify potential collision threats that may be outside the line of sight or detection range of the vehicle's onboard sensors alone. The LLWNN-based UKF module then processes this rich, multimodal data to provide timely and pertinent collision alerts to the driver. These alerts can warn the driver of impending collisions with distant objects, enabling them to take appropriate evasive action. By implementing this integrated LLWNN-UKF approach leveraging sensor data and V2X communication, we aim to reduce the number of collisions caused by reckless driving, which will lead to a decrease in traffic-related fatalities and injuries.

## 1. Introduction

**Figure 1:**
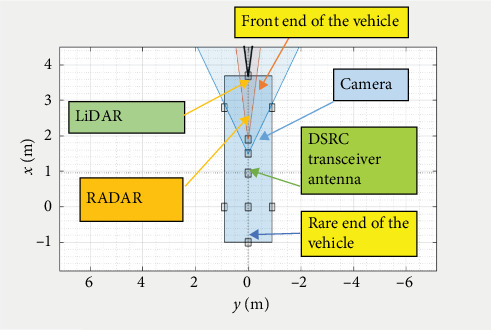
Sensors mounted on the car. LiDAR, light detection and ranging.

Road accidents are the leading cause of mortality for children between the ages of 5 and 29 and result in the deaths of 1.3 million people annually, according to the World Health Organization (WHO). The main risk factors for traffic accidents are speeding, drinking and driving, distracted driving, dangerous driving, unsafe cars, and poor infrastructure [1]. According to the National Highway Traffic Safety Administration's most recent estimates, 42,795 persons died in motor vehicle traffic accidents in 2022. Compared to the 42,939 fatalities reported for 2021, this is a small drop of roughly 0.3%. Nearly 1% more Americans drive in 2022 than in the pandemic's peak year [2]. Some of these accidents were caused by drivers being distracted for a brief period, such as checking their phones or texting. In contrast, others were caused by speeding vehicles running red lights, driving under the influence of alcohol or drugs, or unfavorable road conditions, such as icy or slippery roads or obstacles on the roadway “vehicle-to-everything” (cellular V2X). To address this issue, it is essential to raise awareness among drivers about dangers of inattention and implement measures to improve road safety. This could include the implementation of advanced driver assistance systems (ADASs) technology, which can assist drivers in detecting potential hazards and taking actions to avoid accidents. It is also important to ensure that road infrastructure is well maintained and that traffic laws are strictly enforced to prevent accidents. Accurate information about the area around the cars is essential to guarantee the safety features of an ADAS [3]. Combining data from several onboard sensors, including radio detection and ranging (RADAR), light detection and ranging (LiDAR), and cameras, is one preferred method for accomplishing safety features. The limits of one sensor can be overcome by integrating data from other sensors to get more precise estimations of the condition of nearby objects. The mounting of sensors on the car is shown in Figure 1.

However, this approach using only sensors has data collection range and reliability limitations. Environmental factors such as sensor faults, weather conditions, and solar irradiance can affect sensor data accuracy. Additionally, no data can be obtained if an object is obstructed from view or falls outside the sensor's field of view (FOV). These limitations can compromise the sensor-based perception system's reliability and detection range. It is crucial to increase the perception system's dependability and detection in terms of range and discover a technique to gather data on objects in nonline-of-sight areas to increase road safety further. To get data about items that are not immediately apparent to the sensors, this may include creating new sensor technologies or using alternative techniques. By overcoming these restrictions, it will be feasible to increase the reliability of the data utilized by ADAS and the safety features of on-road vehicles. The following generation of ADASs can be created using wireless vehicular communication networks. Using this technique, drivers may exchange knowledge about their own cars and the environment. Technology “V2X” communication includes vehicle-to-vehicle (V2V), vehicle-to-infrastructure (V2I), and vehicle-to-pedestrian (V2P) communications. V2V communication involves data exchange between two or more vehicles, as opposed to V2I communication, which facilitates data sharing between vehicles and devices on the road. Using a V2P link, vehicle and pedestrians exchange data. Integration of V2V and V2I technology has been found to help prevent ~80% of all auto accidents.

**Figure 2:**
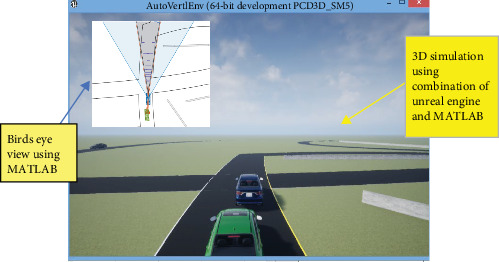
Car-to-car rear impact (MATLAB 3D simulation).

By communicating information about the condition of the roads, the flow of traffic, and potential threats, this sort of communication can help drivers make more informed decisions, avoid collisions, and improve overall road safety.  Figure 2 shows the car-to-car impacts. Wireless vehicular communication networks and onboard sensors can be used to significantly increase the accuracy and reliability of ADAS, enabling the development of future transportation systems that are safer and more efficient. Rear collisions between cars are one of the incidents that occur most frequently on highways due to driver inattention or error. On the open road, at moderate to higher speeds, similar accident situations happen when a motorist is preoccupied and may fail to see that the traffic in front of him has stopped, is coming to a halt, or is going at a slower pace.

**Figure 3:**
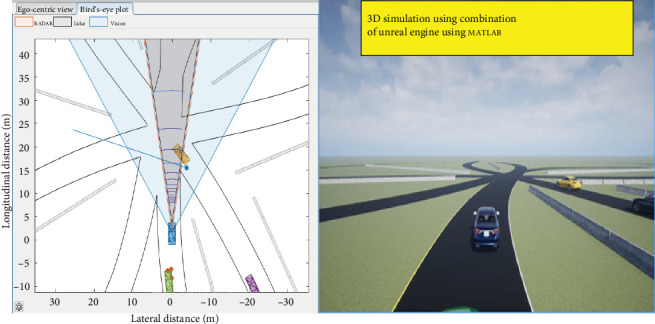
Overview of sensors.

The more complicated nature of the road layout and the perception, judgment, and dynamic maneuvering necessary to properly navigate safely through other vehicles might provide challenges to drivers.  Figure 3 shows the sensor placements. This research proposes a method for predicting vehicle trajectories and issuing collision warnings by fusing data from multiple sensors and V2X communications. Using a combination of sensors and V2X communication can significantly enhance the performance of collision warning systems. A further benefit of V2X communication is that it can provide information about other cars' intentions, such as whether they are turning or changing lanes, allowing for predicting their future paths and reducing false alerts. One challenge is the integration of different data sources and the development of a unified estimation algorithm that can handle various types of data. Another challenge is the reliability of the V2X communication, which can be affected by network congestion or signal interference. Additionally, the accuracy of the V2X communication depends on the penetration rate of V2X-enabled vehicles, which may be limited in some areas. To increase the precision and timeliness of collision alerts in challenging driving situations, a local linear wavelet neural network (LLWNN) based unscented Kalman filter (UKF) is used to estimate vehicular collisions utilizing a fusion of multisensor and V2X data.

We are motivated to work on the potential impact of developing a collision alert system. Our objective is to determine how this system can significantly contribute to avoiding collisions by alerting drivers before a possible accident and improving overall road safety. The aim of utilizing V2X communication, sensor fusion, and applying a suitable filter and neural network for collision estimation is to boost the precision and dependability of the vehicle's perception system and enhance road safety in general. By integrating information from V2X communication and onboard sensors, the perception systems can compensate for each other's deficiencies and achieve a more precise and vigorous perception of the vehicle's surrounding environment. A proper filter can integrate and process the data from both sources, distinguishing between reliable and unreliable data sources and generating accurate collision estimations based on the combined data. This can significantly reduce false alarms and missed detections, resulting in a more reliable and robust collision avoidance system. Combining V2X communication and sensor fusion with a proper filter and neural network can significantly improve collision avoidance and road safety. The perception capabilities of onboard sensors like RADAR, LiDAR, and cameras can be considerably enhanced by V2X communication, leading to a more precise, robust, and comprehensive view of the vehicle's surroundings. The two types of data can complement each other and compensate for each other's shortcomings by integrating data from onboard sensors and V2X communication. The significance of using a LLWNN-based UKF for vehicular collision estimation using the fusion of multisensors and V2X communication data lies in its potential to improve the accuracy and timeliness of collision warning in complex driving scenarios. This approach can provide several benefits over traditional collision warning systems that rely on only a single sensor or communication technology, such as RADAR, camera, LiDAR, or V2X. This research proposes a method for predicting vehicle trajectories and issuing collision warnings by fusing data from multiple sensors and V2X communications. To improve the perception capabilities and reliability of onboard sensors, a wavelet neural network (WNN)–based UKF approach is employed for high-level fusion of RADAR, LiDAR, camera, and V2X communication data.

First, the LLWNN-based UKF approach can fuse data from multiple sensors, such as cameras, LiDAR, RADAR, and V2X communication, providing a more comprehensive and accurate representation of the surrounding vehicles and the system's state. This can help to reduce false alarms and improve the reliability of the collision warning system. Second, the LLWNN-based UKF approach can model the nonlinear relationship between the sensor and V2X data and the state of the system using an LLWNN, which can improve the accuracy of the state estimation and reduce the impact of noise and outliers in the data. Third, the LLWNN-based UKF approach can adapt the smoothing parameters of the UKF based on the operating conditions of the vehicle using the LLWNN, which can improve the responsiveness and accuracy of the state estimation. Finally, the LLWNN-based UKF approach can generate accurate and timely collision warnings based on the estimated state of the system, which can help prevent collisions and improve the safety of the driving environment.

The research contributions are summarized as follows:

1. The method combines the advantages of the UKF and LLWNN to overcome the limitations of traditional collision estimation techniques.
2. The proposed approach can precisely predict the likelihood of a collision and offer early warning of potential collisions, which can significantly improve the safety of autonomous vehicles and reduce the risk of accidents.
3. The method is flexible and can be adapted to different types of sensors and environments, and it can also handle noisy and incomplete sensor data, making it suitable for real-world applications.
4. The UKF-based WNN for collision estimation is a promising approach that has the potential to improve the safety and performance of autonomous vehicles, requiring further research and development to fully explore its capabilities and applications.

Overall, the research demonstrates the effectiveness of the LLWNN-based UKF approach for collision warning systems by fusing data from multiple sensors and V2X communication. The approach can provide more accurate and timely collision warnings, contributing to developing safer and more reliable intelligent transportation systems.

## 2. Related Work

Baek et al. [3] proposed an advanced vehicle collision warning system that leverages multisensors and wireless vehicular communications. The system uses the extended Kalman filter (EKF) to predict the trajectories of remote targets and alert the driver before a potential collision. The primary objective of this system is to minimize fatalities and injuries resulting from driver inattention. The article highlights the significance of ADASs technology in enhancing road safety. By adopting a sensor fusion approach, the system overcomes individual sensors' functional and environmental limitations, resulting in more accurate estimates of the state of the surrounding objects. Xiang et al. [4] focus on a multisensor fusion perception technique for blind spot warning in cooperative vehicle infrastructure systems. The approach combines camera semantic information with range information from LiDAR to obtain object type and location information. The real-time sensing data is transmitted to nearby vehicles using 5G and V2X technology to provide advanced warning of blind spots. Naeem et al. [5] mentioned the importance of the V2X communication protocol in improving road safety and traffic flow and reducing environmental impact. It also highlights the use of vehicular ad-hoc networks to enhance the services of cooperative intelligent transportation systems. Finally, the article concludes by emphasizing the potential of V2X communication modeling and computer simulation through VANET technology. Senel et al. [6] described a modular and real-time capable multisensor fusion framework that enables environmental perception in smart traffic applications. The framework utilizes a combination of classical data fusion algorithms and can be adapted to accommodate different types and numbers of sensors. Kim et al. [7] proposed a collision avoidance algorithm that employs the Kalman filter (KF) to predict states and plan an optimal path while accounting for uncertainty in avoidance scenarios based on the number of ships. An UKF was utilized to predict the state variables of the ships, and the positions of the ships over a specific time are estimated as a predictive probability. Gandy [8] proposed a system ranging from simple backup cameras to sophisticated stereo cameras used to precisely measure objects in three dimensions. Fusing a camera sensor with a range-detecting sensor, such as a RADAR or LiDAR sensor, is a frequent practice. By doing this, a “complementary” pair is formed, which is helpful for precisely detecting the surroundings of the vehicle [9]. RADAR sensors are an established technology that transmits and receives signals using radio frequencies (Rfs) to determine the range and speed of a target. Automotive RADAR frequently uses the 24, 77, and 79 GHz frequency ranges [10]. RADAR sensors perform better in range and velocity measurement than other car sensors, sustain performance under challenging weather conditions like rain and fog, and are often less costly than many LiDAR sensors [11]. In RADAR-based applications, filtering is typically accomplished using a Bayesian filter. The KF is a specific type of Bayesian filter that is commonly used for tracking applications. However, vehicle motion is often nonlinear in automotive applications, necessitating a nonlinear model. A constant turn rate and velocity (CTRV) motion model is commonly used to represent vehicle movement, as described in [12]. The conversion is not linear when the Cartesian kinematic state space is transformed into the polar measurement space, as stated by Manjunath et al. [11]. The LiDAR sensor works by emitting pulses of light and determining distances based on the time it takes for the time of flight (ToF). In the automotive sector, there are three types of LiDAR sensors: rotary-based mechanical LiDAR, scanning solid-state LiDAR, and full solid-state LiDAR [13]. ADASs sensors have a limited detection range and can only detect situations within nonblind spot areas, making them inadequate for detecting obstacles behind obstacles. However, this limitation can be overcome with V2X systems, which have been identified as a key technology for autonomous driving [14]. V2X communication brings a wide spectrum of applications to semi and fully autonomous vehicles. The Traffic Light Assist [8, 15] asssists the drivers anticipate the traffic light changes to enhance the safety and convenience. Platooning [16] is another technology that utilizes V2V messages to give string stability to a line of vehicles.

V2X communication is typically categorized into dedicated short-range communications (DSRCs) and cellular-V2X (C-V2X). DSRC uses the IEEE 802.11p wireless protocol for local transmission and reception of basic safety messages (BSMs), signal timing and phasing (STaP) messages, and map messages. In ideal environments, DSRC radios can communicate with vehicles in the ranges of 3–5 km [17]. DSRC is used for low-latency applications such as intervehicle communication. In areas without 5G cellular coverage, vehicles with DSRC radios can create an ad hoc network [18]. C-V2X leverages 5G network technologies and is standardized in 3rd Generation Partnership Project (3GPP) release 15. Green navigation [19] uses C-V2X to receive traffic information to optimize navigation throughout a city, improving fuel efficiency and avoiding highly congested areas. Optimal vehicle communication uses a hybrid V2X architecture. The hybrid V2X architecture uses DSRC and C-V2X radios to benefit both systems [20]. A robust approach used to estimate the state of a system with nonlinear dynamics and non-Gaussian noise is the UKF [21]. When the measurement variance is known, the KF can determine the state of a dynamic system from a dataset containing measurement noise. Although the particle filter (PF) may be used to solve nonlinear problems, it requires many sample points, making the computation exceedingly difficult. An UKF-based algorithm rebuilds the vehicle trajectory for the signalized junction in response to the aforementioned problems [22]. There are various benefits to collision warning systems when a WNN [5] is used to filter the output of a UKF that combines information from both V2X and sensors installed on the vehicle. This can aid in removing noise and outliers that may be present in the sensor and V2X data, improving the state estimation's accuracy. The effects of rain, snow, fog, and hail on the performance of self-driving cars. Rain and snow, for example, reduce the car's ability to detect obstacles in its path, while fog can impair its ability to maintain lane positioning [23]. While cameras have been used to detect objects like vehicles and pedestrians, they can be computationally expensive and struggle with variations in vehicle appearance, dynamic backgrounds, and disturbances in tracking. The proposed approach is intended to address the limitations of traditional sensors and improve collision avoidance in autonomous vehicles. However, it also has a limitation in that it relies solely on motion features in driving videos, which may not always be sufficient to identify potential collision events accurately. The proposed approach may only be applicable in some driving scenarios. van Ratingen et al. [24] described a fusion sensing approach that combines roadside cameras and LiDAR for blind spot warning applications. The proposed method utilizes semantic information from 2D image object detection and 3D point cloud data from LiDAR to enhance the accuracy of detecting traffic participants, such as pedestrians and vehicles. Yamada et al. [25] proposed an optimal trajectory generation scheme for autonomous vehicle overtaking to enable smooth and safe maneuvers in various traffic conditions. The scheme uses an optimal predictive control approach to minimize driving costs while limiting collision risks. The evaluation is limited to simplified traffic scenarios. The paper focuses on evaluating the scheme in relatively simple traffic scenarios. Bakibillah et al. [26] proposed a car-following scheme based on model predictive control (MPC) to improve traffic flow behavior, particularly in stopping and speeding up of individual vehicles in dense urban traffic under a connected vehicle (CV) environment. The scheme uses V2V communication to predict the future states of the preceding vehicle and compute the control input to minimize costs related to speed deviation, control input, and unsafe gaps. The proposed scheme is entirely dependent on the availability and reliability of V2V communication between vehicles. This could be a significant limitation, as the performance of the scheme may be severely impacted by communication failures, delays, or poor connectivity, especially in areas with low penetration of CVs. Bakibillah et al. [27] presented a bilevel RCS framework comprising vehicle clustering and combinatorial optimization for sequencing and merging of CAVs at roundabouts. While the proposed optimization-based approach shows potential for improving traffic flow, the oversimplified vehicle dynamics model, scalability concerns for high traffic volumes, and lack of robustness to sensor errors and communication delays limit the practical applicability. Furthermore, the study does not address the integration of the RCS with other traffic management systems or consider human factors related to user acceptance of the automated merging system. Fitriyati et al. [28] used the EKF to forecast the rate of change in tumor, healthy host, and effector immune cells in the Itik-Banks model. They linearized the nonlinear model using a first-order Taylor series to construct a new state space representation. Numerical simulations showed strong alignment between EKF predictions and actual data, particularly for tumor and healthy host cells. However, the oscillatory nature of effector immune cells posed a challenge. If the researchers had used the UKF instead of the EKF, the UKF's better handling of nonlinearity could have led to improved accuracy, especially for the oscillatory effector immune cells. The performance would still depend on the model accuracy and data availability.  Table 1 shows the literature review summary.

## 3. Methodology

This study considered different types of sensors, and a multisensor approach was adopted to enhance the detection and tracking capabilities. In particular, information from RADAR, LiDAR, camera, and V2X communication was combined at a high level to forecast the trajectories of close targets and send the driver proper warnings to avoid potential collisions. Software tools such as MATLAB/R2022b, car-Sim2017, and Concept-Draw schematic were used in this study project. Engineers and scientists use MATLAB's potent programing tool to research and create intricate systems and products. It offers a wide variety of mathematical and graphical capabilities for data analysis, image processing, and control system design. A piece of software called Car-Sim2017 is used to simulate and examine vehicle dynamics. Engineers can simulate and test various vehicle designs and driving situations, such as acceleration, braking, and handling characteristics. Concept-Draw diagram is a flexible drawing program with a sizable object library, templates, and a full complement of drawing tools. Technical diagrams, flowcharts, and other types of visual representations of intricate systems and procedures are frequently made using it.

### 3.1. System Design

**Figure 4:**
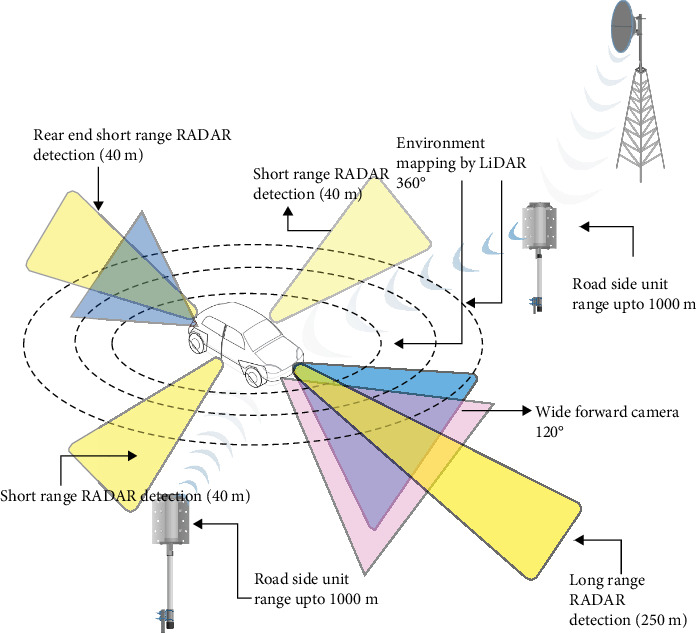
Overview of the system.

The proposed vehicle collision warning system includes a multiphase structure. The camera provides a relative lateral and longitudinal location, and the host vehicle employs RADAR and LiDAR sensors to determine the relative range and azimuth. Additionally, GNSS measurements and dynamic data from distant objects, like speed and yaw rate, are acquired using a DSRC transmitter. Data from each sensor are analyzed using an UKF technique to reduce measurement noise and estimate the state and error covariance at each time step. The UKF is commonly employed to determine the state of the surrounding environment and forecast the future trajectories of other vehicles. However, due to the highly nonlinear driving scenarios, more than the UKF is required. The LLWNN can address these limitations as a postprocessing step after the UKF. The LLWNN can further refine the prediction of target position and velocity by learning from the historical data and detecting any patterns or trends in the data that the UKF may have missed. By combining the UKF with the LLWNN, a more precise and resilient estimation of the state of the surrounding environment can be achieved, potentially resulting in superior collision warning and avoidance systems.  Figure 4 shows the overview of the system.

**Figure 5:**
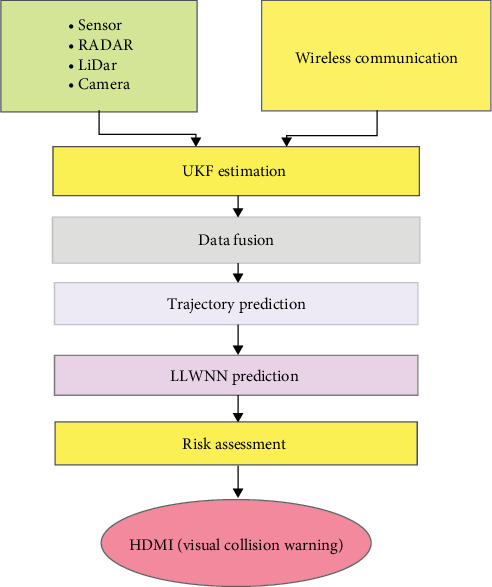
Proposed system block diagram. LiDAR, light detection and ranging; LLWNN, local linear wavelet neural network; RADAR, radio detection and ranging; UKF, unscented Kalman filter.

In summary, integrating the WNN with the UKF in vehicle collision estimation can enhance the system's reliability and efficacy. Subsequently, the system generates suitable visual and auditory alerts to the driver based on the time to collision (TTC) estimate, with the human–machine interface (HMI) presenting warning information in four different threat levels. The proposed system utilizes diverse sensors and advanced algorithms to identify and prevent potential collisions, primarily improving driver safety. The proposed block diagram is shown in Figure 5.

To analyze the advantages of integrating V2X communication devices into today's automobiles in terms of road safety, we selected onboard sensors based on those that have already been utilized in vehicles currently in production:

1. The system gathers data from various sensors, including RADAR, LiDAR, and camera.
2. These sensor data are passed through a high-level data fusion technique to integrate the information from the different sensors.
3. The output of the high-level data fusion is then combined with the wireless communication data and fed into the UKF estimation module.
4. The UKF estimation module further processes the fused data to provide an optimized estimate of the vehicle's state.
5. The output of the UKF estimation is used for trajectory prediction, which forecasts the future trajectory of the vehicle.
6. The trajectory prediction output is then used as input to the LLWNN prediction module, which provides further refinement and prediction of the vehicle's trajectory.
7. The results from the LLWNN prediction are then fed into the risk assessment module, which evaluates the potential collision risks based on the predicted trajectory.
8. Finally, the risk assessment output is used to generate the HDMI (visual collision warning) to alert the driver of any potential collisions.

RADAR, cameras, and LiDAR are the sorts of sensors used in modern automobiles to provide ADAS functions including forward collision warning (FCW) and automated emergency braking (AEB).  Table 2 shows the sensor specifications.

### 3.2. Sensor Perception Models

Due to the physical measuring principles involved, each type of sensor has a unique combination of advantages and disadvantages. LiDAR can detect distances very accurately, but it has a lower spatial resolution than high-definition cameras, which has a negative impact. More information is needed for object recognition and categorization, particularly at a long range [29]. On the contrary, while RADAR often has a lower spatial resolution, it is less susceptible to adverse weather conditions and is not restricted in a range like LiDAR. To mitigate the shortcomings of individual sensors and enhance the reliability of the perception process in the scenarios mentioned, a fusion approach involving sensors and V2X communication is recommended. In this research, we used internal sensors, particularly cameras, LiDAR, and RADAR, in conjunction with V2X to generate data. The MATLAB Driving Scenario Tool Box was used for this purpose. Due to their affordability and high-resolution data output, cameras are frequently incorporated into automobiles for various purposes, including ADAS like emergency braking, object lane, and traffic sign detection. Furthermore, cameras are utilized in HMIs in moving cars and to enhance the detection capabilities of other sensors such as LiDAR and RADAR [29].

#### 3.2.1. V2X Communications

**Figure 6:**
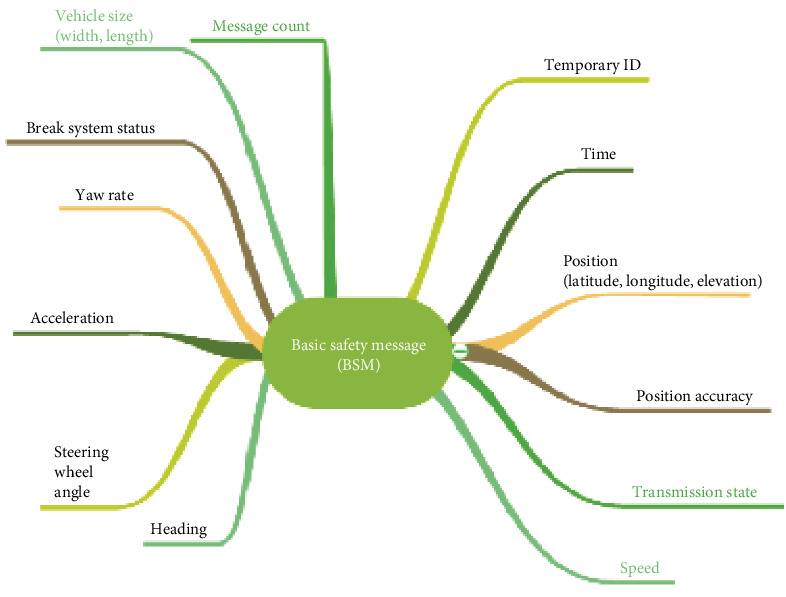
Basic safety message format.

**Figure 7:**
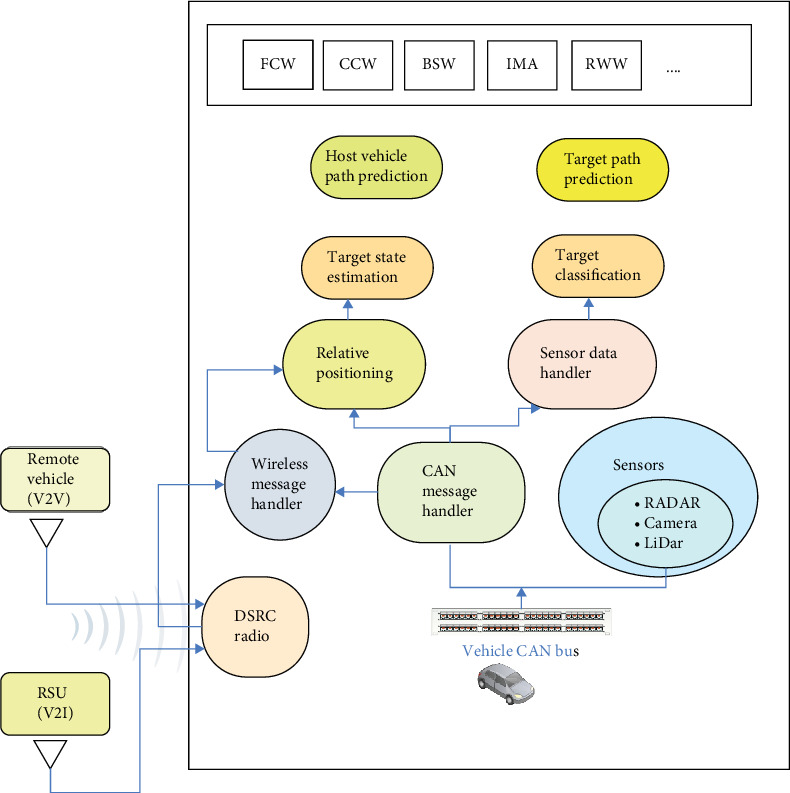
The process of collection of data from different sources. BSW, blind spot warning; CCW, cross-collision warning; FCW, forward collision warning; IMA, intersection movment assistance; RWW, road works warning.

Along with the sensors mentioned in the preceding section, the host vehicle in this study also has a DSRC antenna to enable V2X connection. This enables the host vehicle to communicate BSMs up to a distance of 1000 m with nearby remote cars. The BSMs have a duration of 100 ms and are transmitted over the control channel (CCH). The host car can receive information about the nearby traffic and utilize it to enhance its own safety and driving performance by exchanging BSMs with other vehicles.  Figure 6 depicts the format for basic safety. The collection of data from different sources is shown in Figure 7.

### 3.3. Data Fusion

**Figure 8:**
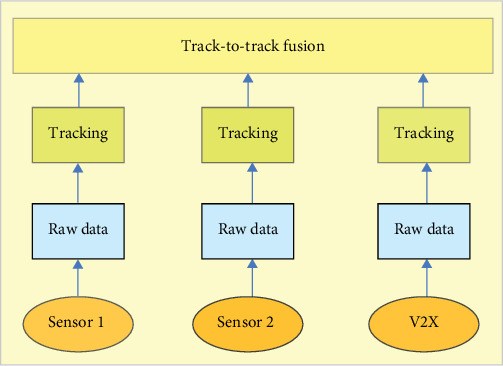
High-level fusion system architecture. V2X, vehicle-to-everything.

High-level data from diverse sources are combined using a track-to-track fusion technique. In a high-level fusion system, data processing for each sensor system is carried out independently at the sensor level. Each sensor system generates one or more tracks based on its observations, and a track-to-track fusion algorithm combines the state estimates from several sensor tracks. The high-level fusion of diverse sensors has been successfully used in numerous research on automotive applications and offers benefits including spatial and temporal alignment, modularity, and reduced communication overhead.  Figure 8 shows a diagram of the high-level fusion system architecture [6].

It utilizes a fusion technique called high-level fusion technique. High-level sensor fusion is an architecture where each sensor independently performs object detection and tracking, and the fusion module then associates and combines these sensor-level object tracks. This is in contrast to low-level fusion, where the raw sensor data are combined before any object detection or tracking is performed. The key advantage of high-level fusion is its modularity and encapsulation of sensor-specific details. By keeping the sensor-level processing independent, the fusion module can operate on a more abstract level without needing to understand the intricacies of each individual sensor. This makes the overall system design simpler and more flexible, as sensors can be added, removed, or updated without extensively modifying the fusion logic.

### 3.4. Proposed UKF Method

For extremely nonlinear systems, the UKF [22] is an improved version of the EKF. The UKF uses a deterministic sampling approach called the unscented transform to approximate the mean and covariance of the state distribution. Here, we present the steps to update the equations for the UKF.

The state vector at time step *k* is still defined by the following equation:

(1) $$\begin{array}{c} x_{k}={\left[x_{k}y_{k}v_{x,k}v_{y,k}\right]}^{T}. \end{array}$$

To implement the UKF, we first need to generate sigma points. The sigma points are chosen to represent the state distribution. The sigma points at time step k−1 are given by the following equation:

(2) $$\begin{array}{c} x_{k-1/k-1}=\left[x_{k-1/k-1,}x_{k-1/k-1}+{\left(\sqrt{\left(n+\lambda \right)}p_{k-1/k-1}\right)}^{i},x_{k-1/k-1}-{\left(\sqrt{\left(n+\lambda \right)}p_{k-1/k-1}\right)}^{i}\right]ith, \end{array}$$

where *λ* is a scaling parameter, *n* is the dimension of the state vector, *p*_*k*−1/*k*−1_ is the state covariance matrix, and ${\left(\sqrt{\left(n+\lambda \right)}p_{k-1/k-1}\right)}^{i}$ represents the *i*th column of the matrix square root. The next step is to propagate the sigma points through the nonlinear system dynamics:

(3) $$\begin{array}{c} x_{k/k-1}=f\left(x_{k-1/k-1}\right), \end{array}$$

where *f* is the nonlinear state transition function.

Now, compute the predicted state mean and covariance: *x*_*k*/*k*−1_:

(4) $$\begin{array}{c} \overset{\wedge }{x}=\sum \left(W_{m_{j}}\times X_{k_{i}/k-1}\right), \end{array}$$

(5) $$\begin{array}{c} P_{k/k-1}=\sum \left(W_{c_{j}}\times \left(X_{k_{i}/k-1}-x_{k/k-1}\right){\left(X_{k_{i}/k-1}-x_{k/k-1}\right)}^{T}\right)+Q, \end{array}$$

where *W*_*m*_*i*__ and *W*_*c*_*j*__ are the weights for the mean and covariance.

Next, transform the propagated sigma points into the measurement space:

(6) $$\begin{array}{c} Z_{k}=h\left(X_{k/k-1}\right), \end{array}$$

where *h* is the nonlinear measurement function.

Compute the predicted measurement mean and covariance:

(7) $$\begin{array}{c} \overset{\wedge }{z_{k}}=\sum \left(W_{m_{i}}\times Z_{K_{i}}\right), \end{array}$$

(8) $$\begin{array}{c} P_{zz}=\sum \left(W_{c_{i}}\times \left(Z_{k_{i}}-\overset{\wedge }{z_{k}}\right){\left(Z_{k_{i}}-\overset{\wedge }{z_{k}}\right)}^{T}\right)+R. \end{array}$$

Calculate the cross-covariance matrix:

(9) $$\begin{array}{c} P_{xz}=\sum \left(W_{c_{j}}\times \left(X_{k_{j}/k-1}-x_{k/k-1}\right){\left(Z_{k_{j}}-\overset{\wedge }{z_{k}}\right)}^{T}\right). \end{array}$$

Now, compute the Kalman gain:

(10) $$\begin{array}{c} K_{K}=P_{jx^{z}}\times {\left(P_{zz}\right)}^{-1}. \end{array}$$

Finally, update the state and covariance:

(11) $$\begin{array}{c} {\overset{\wedge }{x}}_{k/k}={\overset{\wedge }{x}}_{k/k-1}+K_{k}\times \left(Z_{k_{j}}-\overset{\wedge }{z_{k}}\right), \end{array}$$

(12) $$\begin{array}{c} P_{k/k}=P_{k/k-1}-K_{k}\times P_{zz}\times {K_{k}}^{T}. \end{array}$$

#### 3.4.1. Trajectory Prediction and Risk Assessment

**Figure 9:**
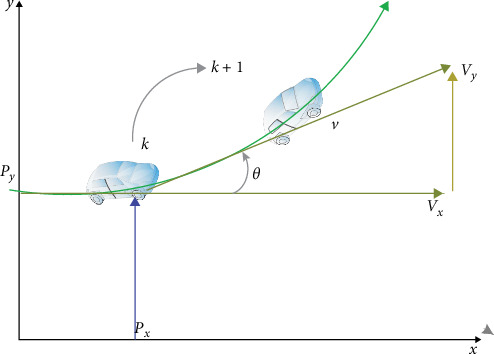
Trajectory of a prediction of the vehicle.

In this study, our focus is on a dynamic object that exhibits nonlinear motion described by the CTRV model [30, 31]. This model accounts for the possibility of the object moving in a straight line as well as with a CTRV magnitude, as shown in Figure 9:

1. The vector that represents the state of a CTRV model:
  (13) $$\begin{array}{c} x={\left[p_{x}p_{y}v\theta \beta^{\prime }\right]}^{T}. \end{array}$$
• *v*: speed, which refers to the magnitude.
• *θ*: yaw angle, which denotes the orientation.
• *β* ^″^: rate of change in yaw angle.

(14) $$\begin{array}{c} x^{\prime }={\left[p_{{\text{⁣}}^{\prime }}p_{{\text{⁣}}^{\prime }}v^{\prime }\beta^{\prime }\beta^{\prime \prime }\right]}^{T}={\left[v\cdot \cos{\left(\theta \right)}^{\text{⁣}}v\cdot \sin{\left(\theta \right)}^{\text{⁣}}0^{\text{⁣}}\beta^{\prime }0\right]}^{T}. \end{array}$$

2. Time difference: Δ*t* = *t*_*k*+1_ − *t*_*k*_.
3. Process model (predicts the state at time step *k* + 1):

(15) $$\begin{array}{c} x_{k+1}=f\left(x_{k},v_{k}\right)=x_{k+1}=x_{k}+\left[\begin{array}{c} \frac{vk}{\beta_{k}}\left(\sin\left(\theta_{k}+{\dot{\beta }}_{k}\Delta t\right)-\sin\left(\theta_{k}\right)\right)\\ \frac{vk}{\beta_{k}}\left(-\cos\left(\theta_{k}+{\dot{\beta }}_{k}\Delta t\right)+\cos\left(\theta_{k}\right)\right)\\ 0\\ {\dot{\beta }}_{k}\Delta t\\ 0 \end{array}\right]+\left[\begin{array}{c} \frac{1}{2}{\left(\Delta t\right)}^{2}\cos\left(\theta_{k}\right)\cdot v_{a,k}\\ \frac{1}{2}{\left(\Delta t\right)}^{2}\sin\left(\theta_{k}\right)\cdot v_{a,k}\\ \Delta t\cdot v_{a,k}\\ \frac{1}{2}{\left(\Delta t\right)}^{2}\cdot v_{\beta ,k}\\ \Delta t\cdot v_{\vec{\beta },k} \end{array}\right]. \end{array}$$

Note: We should be careful when ${\dot{\beta }}_{k}=0$, to avoid divison by 0 (this is situation when the yaw angle is not changed, the car is going straight) if ${\dot{\beta }}_{k}$ is zero:

(16) $$\begin{array}{c} x_{k+1}=x_{k}+\left[\begin{array}{c} v_{k}\cos\left(\theta_{k}\right)\Delta t\\ v_{k}\sin\left(\theta_{k}\right)\Delta t\\ 0\\ \beta_{k}\Delta t\\ 0 \end{array}\right]+\left[\begin{array}{c} \frac{1}{2}{\left(\Delta t\right)}^{2}\cos\left(\theta_{k}\right)\cdot v_{a,k}\\ \frac{1}{2}{\left(\Delta t\right)}^{2}\sin\left(\theta_{k}\right)\cdot v_{a,k}\\ \Delta t\cdot v_{a,k}\\ \frac{1}{2}{\left(\Delta t\right)}^{2}\cdot v_{\beta ,k}\\ \Delta t\cdot v_{\vec{\beta },k} \end{array}\right]. \end{array}$$

Consider only deterministic part:

(17) $$\begin{array}{c} x_{k+1}=f\left(x_{k}\right)=x_{k}+\int_{t_{k}}^{x+1}{\left[v\left(t\right)\cdot \cos\left(\theta \left(t\right)\right)v\left(t\right)\cdot \sin\left(\theta \left(t\right)\right)0\dot{\beta }0\right]}^{T}dt, \end{array}$$

(18) $$\begin{array}{c} x_{k+1}=x_{k}+\left[\begin{array}{c} \int_{t_{k}}^{t_{k+1}}v\left(t\right)\cdot \cos\left(\theta \left(t\right)\right)dt\\ \int_{t_{k+1}}^{t_{k+1}}v\left(t\right)\cdot \sin\left(\theta \left(t\right)\right)dt\\ 0\\ {\dot{\beta }}_{k}\Delta t\\ 0 \end{array}\right]=x_{k}+\left[\begin{array}{c} v_{k}\int_{t_{k}}^{t_{k+1}}\cos\left(\theta_{k}+{\dot{\beta }}_{k}\cdot \left(t-t_{k}\right)\right)dt\\ v_{k}\int_{t_{k+1}}^{t_{k+1}}\sin\left(\theta_{k}+{\dot{\beta }}_{k}\cdot \left(t-t_{k}\right)\right)dt\\ 0\\ \beta_{k}\Delta t\\ 0 \end{array}\right], \end{array}$$

(19) $$\begin{array}{c} x_{k+1}=x_{k}+\left[\begin{array}{c} \frac{vk}{\beta_{k}}\left(\sin\left(\theta_{k}+{\dot{\beta }}_{k}\Delta t\right)-\sin\left(\theta_{k}\right)\right)\\ \frac{vk}{\beta_{k}}\left(-\cos\left(\theta_{k}+{\dot{\beta }}_{k}\Delta t\right)+\cos\left(\theta_{k}\right)\right)\\ 0\\ {\dot{\beta }}_{k}\Delta t\\ 0 \end{array}\right]. \end{array}$$

• The random component (vector of noise): $v_{k}=[\begin{array}{ll} v_{a,k} & v_{\beta ,k} \end{array}{]}^{T}$.
• Noise in longitudinal acceleration: *v*_*a*,*k*_ ~ *𝒩*(0, *σ*_*a*_^2^).
• Noise in yaw acceleration: *v*_*β*,*k*_ ~ *𝒩*(0, *σ*_*β*_^2^).

Let us make the assumption that the longitudinal and yaw acceleration noise remain constant between steps *k* and *k* + 1. Additionally, let us assume that the vehicle is moving in a straight line, so that we can calculate the longitudinal and yaw accelerations separately (this approximation will be valid as long as the yaw rate remains within a reasonable range):

(20) $$\begin{array}{c} f\left(v_{k}\right)=\left[\begin{array}{c} \frac{1}{2}{\left(\Delta t\right)}^{2}\cos\left(\theta_{k}\right)\cdot v_{a,k}\\ \frac{1}{2}{\left(\Delta t\right)}^{2}\sin\left(\theta_{k}\right)\cdot v_{a,k}\\ \Delta t\cdot v_{a,k}\\ \frac{1}{2}{\left(\Delta t\right)}^{2}\cdot v_{\beta ,k}\\ \Delta t\cdot v_{\vec{\beta },k} \end{array}\right]. \end{array}$$

Compensating for ego motion is crucial for accurate perception when the sensor carrier undergoes changes in speed or orientation. Even a slight change in orientation can lead to significant variations in the lateral position of an object, depending on its longitudinal distance, leading to negative impacts on object association. Additionally, the estimation accuracy can be severely degraded even when object association is successful since the tracked object state vector variables are relative to the sensor carrier position and orientation [6, 30–32].

#### 3.4.2. Proposed LLWNN

**Figure 10:**
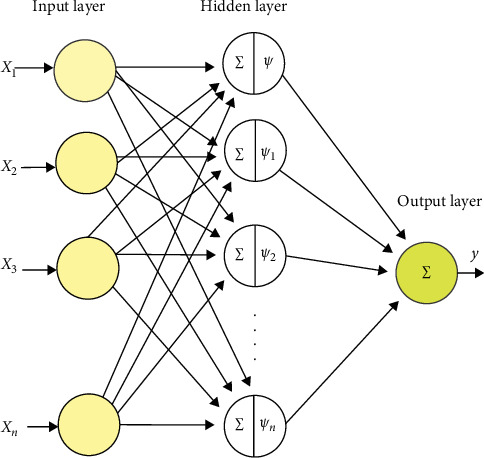
The building of a local linear wavelet neural network.

LLWNNs are artificial neural networks that use wavelet activation functions. Using a local linear approximation, LLWNN can model nonlinear relationships more accurately and efficiently than other types of neural networks. The architecture is shown in Figure 10. The input layer takes the input data as a vector of values x = [*x*_1_, *x*_2_,…*x*_*n*_]. The hidden layer transforms the input data vector into the shifted and scaled shapes of a mother wavelet.

The data point *x*_1_, *x*_2_,…, *x*_*n*_ are inputs and *ψ*_1_, *ψ*_2_,…, *ψ*_*n*_ are the wavelet activation function. The wavelet function used in the model is proposed as a way to capture the local features of the input data by analyzing multiple scales and frequencies. The objective function is given by the following equation:

(21) $$\begin{array}{c} x_{n}=x_{n-1}+\left[\begin{array}{c} \frac{vk}{\beta_{k}}\left(\sin\left(\theta_{k}+{\dot{\beta }}_{k}\Delta t\right)-\sin\left(\theta_{k}\right)\right)\\ \frac{vk}{\beta_{k}}\left(-\cos\left(\theta_{k}+{\dot{\beta }}_{k}\Delta t\right)+\cos\left(\theta_{k}\right)\right)\\ 0\\ {\dot{\beta }}_{k}\Delta t\\ 0 \end{array}\right], \end{array}$$

(22) $$\begin{array}{c} \psi_{n}\left(x\right)=-x\;\exp\left(-\frac{x^{2}}{2}\right), \end{array}$$

(23) $$\begin{array}{c} y_{n}=\sum_{i=1}^{N}\left(w_{i0}+w_{i1}x_{1}+w_{iN}x_{N}\right)\psi_{n}\left(x\right). \end{array}$$

The objective function in machine learning is a function that is optimized during training to minimize the error between the predicted output and the actual output. In regression problems, mean square error (MSE) is a common loss function used to measure the difference between the predicted and actual values.

The MSE is calculated for each data point in the training set. Mathematically, the MSE can be written as follows:

(24) $$\begin{array}{c} \text{MSE}\left(e\right)=\frac{1}{N}\sum_{n=1}^{N}{\left(Y-y_{\text{pred}\sim \text{⁣}}\right)}^{2}, \end{array}$$

(25) $$\begin{array}{c} R^{2}=1-\frac{\sum_{t=1}^{n}{\left(Y-y_{\text{pred}\sim \text{⁣}}\right)}^{2}}{\sum_{t=1}^{n}{\left(Y-y\right)}^{2}}, \end{array}$$

where *N* is the number of data points in the training set, *Y* is the actual output, *y*_pred~⁣_ is the predicted output, *y* is the mean of the actual value, and the summation is taken over all data points in the training set. During training, the objective is to find the model parameters that minimize the MSE. MSE is widely used in machine learning because it is easy to interpret and can be used to compare the performance of different models.

The UKF has several advantages compared to the adaptive EKF:

1. Nonlinearity handling: The UKF is better at handling highly nonlinear systems compared to the EKF. The EKF relies on a first-order Taylor series approximation of the nonlinear functions, which can introduce significant errors, especially for strongly nonlinear systems. In contrast, the UKF uses a set of carefully chosen sample points (sigma points) to capture the true mean and covariance of the state, which provides a more accurate representation of the nonlinear transformations.
2. Easier implementation: The UKF is generally simpler to implement than the adaptive EKF. The UKF algorithm involves the deterministic sampling of the state distribution and the propagation of these sample points through the nonlinear functions, followed by the computation of the mean and covariance. This process is more straightforward than the Jacobian calculation and linearization required for the EKF.
3. Better estimation accuracy: Due to its superior handling of nonlinearities, the UKF often provides more accurate state estimates compared to the EKF, especially for systems with strong nonlinearities.
4. No Jacobian calculation: The UKF does not require the calculation of Jacobian matrices, which can be computationally expensive and prone to numerical instabilities, particularly for complex nonlinear functions.
5. Automatic handling of uncertainties: The UKF automatically captures the effect of input, process, and measurement uncertainties through the propagation of the sigma points, whereas the EKF requires manual linearization and calculation of the uncertainty propagation.
6. Adaptability: The adaptive EKF includes an additional step to adaptively tune the process noise covariance matrix based on the innovation sequence. The UKF can also be made adaptive by incorporating a similar mechanism to adjust the process noise covariance, providing additional flexibility in handling time-varying system dynamics.

It is important to note that the choice between the UKF and the adaptive EKF depends on the specific application, the level of nonlinearity in the system, the available computational resources, and the required estimation accuracy. Both methods have their own strengths and can be effective in different scenarios.

## 4. Results

**Figure 11:**
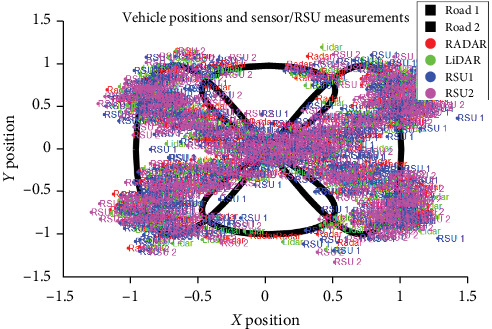
Displays the measurement data collected through sensors and RSUs.

We first create a system simulation where vehicles move through a junction, and different sensors and RSUs measure their positions and velocities. The system is simulated on a 2D plane. Each data point represents a vehicle at a particular time and includes its proper position, velocity, and noisy measurements from different sensors and RSUs. The sensors and RSUs used in the simulation include a RADAR, a LiDAR, and two RSUs. The measurements from these sensors and RSUs are generated by adding random noise to the proper position of the vehicle. The plot represents the position of the cars and the measurements from the sensors and RSUs. The black curve represents the position of the cars, and the colored dots represent the measurements from the sensors and RSUs are shown in Figure 11. The legend indicates which sensor or RSU each dot represents.

This is how the proposed system is tested:

1. Sensor and RSU data are gathered.
2. The UKF estimates the system state for RADAR, LiDAR, RSU1, and RSU2.
3. The estimated state is employed as an input for a LLWNN at each time step.
4. The LLWNN then processes the input and generates an output, which predicts the target variable.
5. The system is then tested in a real-time driving scenario to evaluate its performance in practical applications.

This process can be repeated for each time step in the data set, allowing the neural network to adapt to changes in the system over time. By using real-time data from the sensors and RSUs and combining it with the estimation from the UKF, the neural network can make accurate predictions of the target variable.

### 4.1. Comparison of Nonlinear Filters for State Estimation

**Figure 12:**
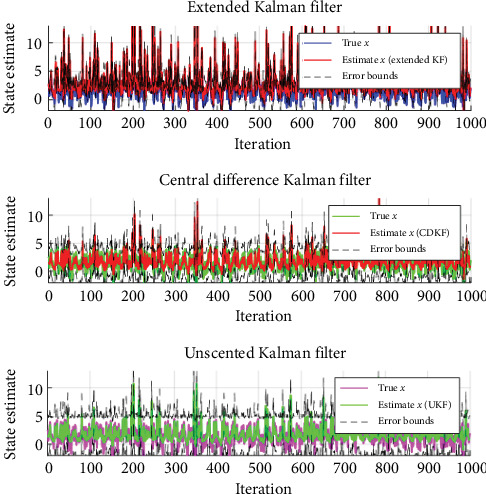
The state estimation performance of different nonlinear filters. CDKF, central difference Kalman filter; KF, Kalman filter; UKF, unscented Kalman filter.

The EKF, central difference Kalman filter (CDKF), and UKF are all nonlinear filtering algorithms that can estimate the state of a system based on noisy measurements. In this case, the system's state was the vehicles' position and velocity, and the measurements would be the noisy positions from the different sensors and RSUs. We have tested using both the EKF and CDKF, which are based on the KF, but they use various techniques to handle the nonlinearities in the system. The EKF linearizes the system dynamics and measurement equations and then applies the KF [25, 26] to estimate the system's state. The CDKF uses a set of sigma points that are symmetrically located around the state's mean to propagate the state's mean and covariance through the nonlinear system dynamics and measurement equations. Compared to these algorithms, the UKF uses a different set of sigma points chosen to capture the mean and covariance of the state accurately. It does not require linearization of the system dynamics and measurement equations. Instead, it propagates the mean and covariance of the state through the nonlinear equations of sigma points. In terms of accuracy, all three filtering algorithms can provide more accurate estimates of the state of the system than the raw sensor measurements, as they consider the uncertainties in the measurements and the dynamics of the vehicles. However, the accuracy of each algorithm can depend on the specific characteristics of the system being modeled, such as the level of nonlinearity and the amount of noise in the measurements shown in Figure 12.

Table 3 shows the MSE values for the three different filtering algorithms. The MSE measures the average squared difference between the estimated value and the true value of a quantity being estimated. In the context of filtering, the MSE represents how well the filter can calculate the true value of a system state given noisy measurements. From the table, it appears that as the nonlinearity increases, UKF has the lowest MSE value, followed closely by the CDKF presented in Table 4. The EKF has a significantly higher MSE value than the other two filters, suggesting it may not perform as well in estimating the true system state.

### 4.2. Using a Single Sensor

**Figure 13:**
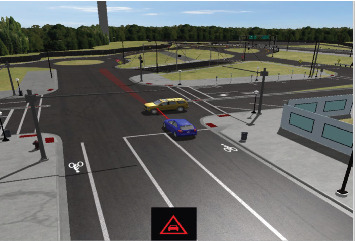
Sensor simulation for a sensor with CarSim17.

Figure 13 demonstrated using CarSim2017 shows that a car's blind spot detection system can generate a warning to the driver by using sensors alone. However, there may be some limitations regarding the timing of the warning. This is because the sensors may not be able to detect all objects in the blind spot, and some objects may only be detected at a later stage. As a result, it may be challenging to provide an early warning to the driver in such situations.

### 4.3. A 2D Simulation Scenarios V2V FCW and CCW (ICW)

**Figure 14:**
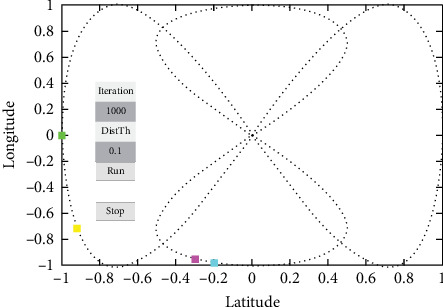
Starting points for the derived scenario.

For the simulation of the proposed method and how well it works, we divided four vehicles into forward-collision scenarios and cross-collision scenarios (CCSs). A set of images is shown for the simulation with timely mannered following the path of the vehicles. There are four vehicles in the derived scenario: two vehicles in purple and cyan going on the same road (road 1) and the other two green and yellow vehicles going the other path on the same road (road 2). The purple car is the ego vehicle (host vehicle). The starting points for the green, yellow, purple, and fourth cars are specified as (−1, 0), (−0.9358, −0.7561), (−0.9713, −0.7729), and (−0.2104, −0.9985), respectively, where the first value in each pair represents the longitude and the second value represents the latitude shown in Figure 14.

#### 4.3.1. V2V Forward-Collision Scenario

**Figure 15:**
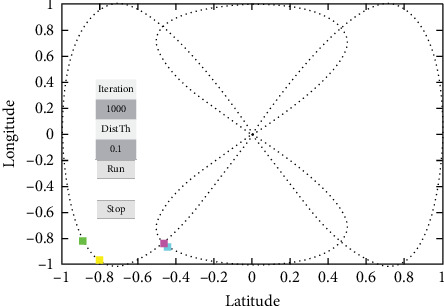
Two cars are approaching each other, there will be a collision if the two cars continue in the same manner.

At some point in time during the simulation, the purple and cyan vehicles started approaching each other, which likely the proposed method would be tested to avoid a collision by warning the driver which is by taking data from sensors and RSUs to the estimation of collision using an UKF-based LLWNN.  Figure 15 shows the two cars approaching each other on the same road at the coordinate of (−0.43956, −0.8623). Using the proposed system, the estimation is done, and a possible collision warning popup on the HDMI for the driver, saying that “Warning!!! Possibly Collision detected.”

**Figure 16:**
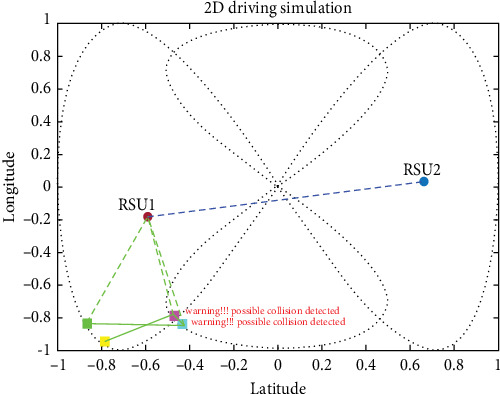
Different sensors are installed in each vehicle and connected through wireless communication.

The drivers then can adjust the speed, in our research simulation, we used that after the possible collision is detected, both the first car and the preceding car increase the speed, and the follower car decreases the speed, as shown in Figure 16.

**Figure 17:**
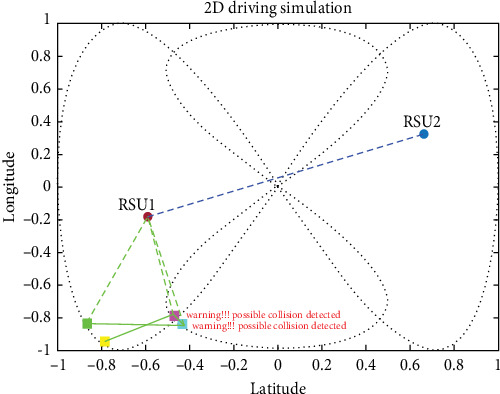
Connectivity of different sensors and wireless communication, which transfers the status of each vehicle to the other.

This connectivity allows for the real-time exchange of information about each vehicle's status and location. By using this information, it is possible to estimate potential collisions and take action to avoid them. As we have tested, the proposed system shows that in the real-world deriving scenario, it is very effective and applicable, which saves the lives of thousands around the world. The proposed system not only has the potential to save lives, but it can also prevent substantial financial losses that would result from accidents and associated economic issues, as shown in Figure 17.

**Figure 18:**
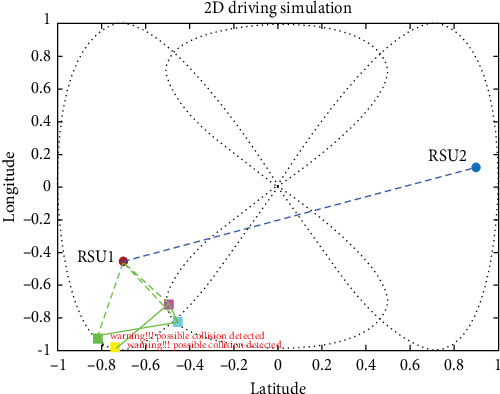
Other vehicles within the range of 1 km.

The proposed system was effective in avoiding collisions in the forward-collision scenario. This indicates that the combination of the UKF, LLWNN, and real-time data from sensors and RSUs was able to accurately estimate potential collisions and take appropriate action to prevent them. Preventing collisions in a forward-collision scenario is particularly important, as these types of collisions can be very dangerous and often result in serious injuries or fatalities. The fact that the proposed system prevented such collisions is a promising sign of its potential effectiveness in real-world driving situations, as shown in Figure 18.

#### 4.3.2. CCS (Intersection Collision Warning)

**Figure 19:**
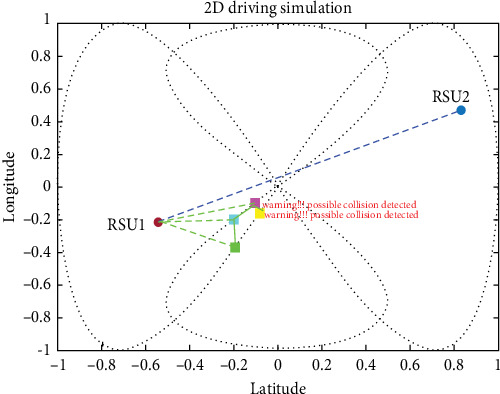
Before reaching the intersection point, the warning generated early before collision using the proposed system.

For CCS, we used four vehicles coming from two different roads, and the proposed system was effective in avoiding collisions in the CCS. Cross-collisions can be particularly difficult to detect and prevent, as they involve vehicles traveling in perpendicular directions and require more complex maneuvers to avoid. In fact, the proposed system could accurately estimate potential collisions and take appropriate action to prevent them in the CCS, indicating its potential effectiveness in real-world driving situations. Integrating the UKF, LLWNN, and real-time data from sensors and RSUs is a promising approach for detecting and preventing collisions in various scenarios, as shown in Figure 19.

**Figure 20:**
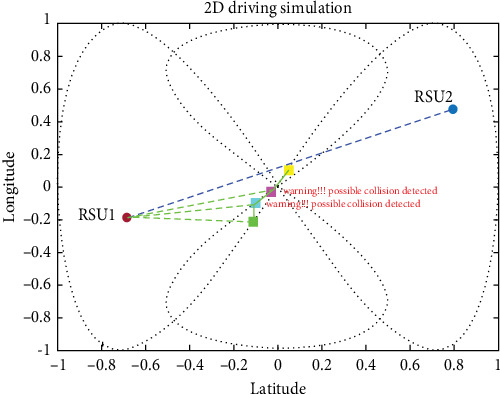
Estimation is done in the complex deriving scenario, which involves blind spot communication in a cross-collision scenario.

**Figure 21:**
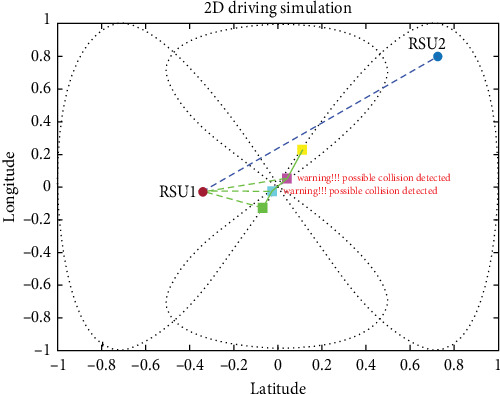
Without collision, when the four vehicles passed the situation.

The proposed system demonstrated its ability to prevent collisions in a complex driving scenario, allowing the four cars to pass through the scenario without any incidents. This success indicates that integrating the UKF, LLWNN, and real-time data from sensors and RSUs is an effective approach to enhancing collision avoidance systems in real-world driving situations, as shown in Figure 20. As real-world driving scenarios can be unpredictable and complex, the proposed system's effectiveness in such a scenario is a promising indication of its potential usefulness in practical applications. However, it is important to note that the proposed system's effectiveness depends on various factors, such as the sensor data's accuracy, the control system's responsiveness, and the complexity of the driving environment.  Figure 21 shows the four vehicles passed the situation without collision.

**Figure 22:**
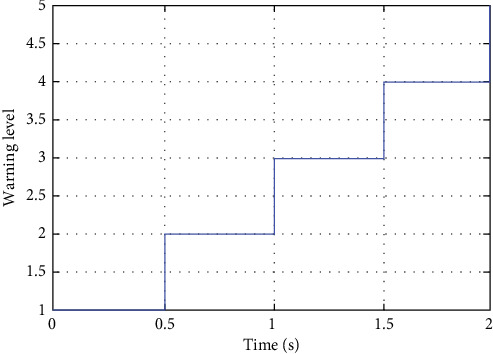
Warning level vs. time using the proposed method.

Figure 22 shows the proposed system providing warnings to the driver at different collision risk levels, with the warning time ranging from less than 2 s to just before the collision. Using step-estimated collision levels may give the driver a better understanding of the collision risk and help them take appropriate action to avoid the collision.

### 4.4. Dataset

A total number of 4430 data is considered for training, testing, and validation. Each neural network model was fed with 70% of the data for training the network. In comparison, the remaining 20% were allocated for testing and 10% for validation, respectively. Table 4 shows the data description, and Table 5 shows the computational time of filters. The sample of data is presented in Table A.1 in Appendix 1.

The computational time for nonlinear filters we used for state estimation at the output of each sensor and RSUs:

Time taken by UKF: 0.001838 s.

Time taken by EKF: 0.0018133 s.

Time taken by CDKF: 0.020520 s.

The computing time of EKF and UKF are almost similar. The EKF is known to perform better for linear or mildly nonlinear systems, as it linearizes the system model around the current state estimate. In contrast, the UKF is designed to handle highly nonlinear systems more effectively, as it uses a deterministic sampling approach (unscented transformation) to capture the true mean and covariance of the transformed variables.

**Figure 23:**
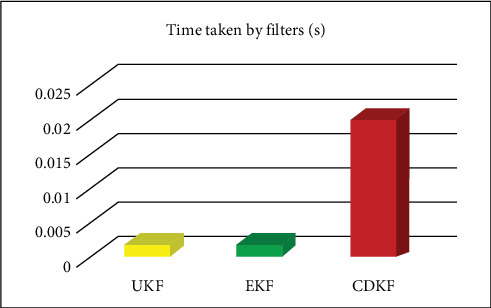
Comparison of computational time of filters. CDKF, central difference Kalman filter; EKF, extended Kalman filter; UKF, unscented Kalman filter.

For the proposed method UKF, initialization of the state and covariance estimates has a complexity of O(*n*^2^). The process model and measurement model functions have a complexity of O(*n*). The number of sigma points used in the filter is 2*n* + 1, so the computational complexity of the UKF per time step is O(*n*^3^) for the matrix operations required to propagate the sigma points through the process and measurement models, and O(*n*^2^) For the Kalman gain computation and state update. The total computational complexity of the UKF for the entire time horizon of length T is O(T*n*^3^). The comparison of computational time is presented in Figure 23.

## 5. Conclusions and Future Scope

A new approach for collision estimation using an UKF-based LLWNN is proposed. The proposed method combines the advantages of the UKF and LLWNN to overcome the limitations of traditional collision estimation methods. The UKF is a nonlinear filtering technique that estimates a system's state based on noisy sensor measurements. The UKF is well-suited for nonlinear systems and can handle uncertain and incomplete data, making it a popular choice for collision estimation in autonomous vehicles. The LLWNN, on the other hand, is a type of neural network that uses wavelet functions as activation functions. The proposed method combines these two technologies to estimate the collision probability and provide early warning of potential collisions. The UKF estimates the system's state based on sensor data, while the LLWNN models the collision probability based on the estimated state. The proposed method offers warnings to drivers at varying collision risk levels, from less than 2 s to just before the collision, by providing step-estimated collision levels. This feature enables drivers to understand the risk of collisions better and take appropriate measures to avoid them. The outcomes of the simulation experiments show that the proposed method performs better in accuracy and efficiency than other methods. The suggested approach can precisely predict the likelihood of a collision and offer early warning of potential collisions, significantly raising the safety of autonomous vehicles and lowering the risk of accidents. In addition, the proposed method is flexible and can be adapted to different types of sensors and environments. It can also handle noisy and incomplete sensor data, making it suitable for real-world applications. One drawback is that the UKF-based LLWNN for collision warning can be sensitive to the initial state estimate and the choice of sigma points in the UKF. Inaccurate estimation can lead to incorrect collision warnings, so careful tuning of UKF parameters and state initialization is crucial for accurate performance. Overall, the proposed UKF-based WNN for collision estimation is a promising approach that can improve the safety and performance of autonomous vehicles. Further research and development are needed to fully explore this method's capabilities and potential applications in various fields. A new model can be developed by exploring different combinations of wavelet functions and neural network architectures and incorporating sensor data into the collision estimation model. Another area of future research could be implementing and testing the proposed method in real-world settings. While the simulation experiments provide a helpful proof-of-concept, further testing and validation in real-world environments would be necessary to evaluate the capabilities and limitations of the method.

## Figures and Tables

**Table 1:** Literature review summary.

References	Title	Proposed method	Finding	Research gap
Beak et al. [3]	Vehicle Trajectory Prediction and Collision Warning via Fusion of Multisensors and Wireless Vehicular Communications	Fusion for multisensors and collision estimation using Kalman filter only	Early collision warning is possible	The estimation only done with a Kalman filter which designed to work in a linear system but the real-life deriving scenario is nonlinear

Xiang et al. [4]	Multi-Sensor Fusion Algorithm in Cooperative Vehicle-Infrastructure System for Blind Spot Warning Chao	Fusion of camera and LiDAR for object detection and sent the data through V2X technology	Collision warning is possible	The detection depends on the camera and LiDAR output and V2X is used to transfer the data, there is no filter, only two sensors used the probability of misleading output is high

Naeem et al. [5]	Vehicle to Everything (V2X) Communication Protocol by Using Vehicular Ad-Hoc Network	Cellular based C-V2X	Collision warning is possible	V2X needs reliable and robust communication infrastructure: • High band, • Low-latency • Secure communication channel • Possible packet data loss is imminent which makes it difficult on dependability of only V2X • Filtering the incoming data should be done even if sensors are used additionally

Kim et al. [7]	Collision Avoidance Based on Predictive Probability Using Kalman Filter	Estimation using Kalman filter	Collision prediction	Kalman filter for collision estimation is that they assume a linear system model with Gaussian noise. The assumption may not hold in all scenarios

Senel et al. [6]	Multi-Sensor Data Fusion for Real-Time Multi-Object Tracking	Using sensor fusion and estimation using unscented Kalman filter	Tracking an object	• Vulnerable to sensor failure • Coverage area is small • Sensitive to environmental conditions • Blind spot coverage

**Table 2:** Sensor specification.

Sensor types	Pixel, frequency, and wavelength	Range (m)	Range accuracy	Angular accuracy	Horizontal FOV	Data update
Camera	640 × 480 pixels	70–100	5% error at 45 m 10% error at 90 m	—	120°	—

RADAR	76–77 GHz frequency band	175–250	0.1−0.5 m	0.1°–0.5°	20°−30°	50–80 ms

LiDAR	905 nm laser wavelength	80	0.1 m	0.25 horizontal resolution	145°	40 ms

**Table 3:** MSE of EKF, CDKF, and UKF.

Filter	MSE
EKF	7.0436
CDKF	0.85529
UKF	0.83137

Abbreviations: CDKF, central difference Kalman filter; EKF, extended Kalman filter; MSE, mean square error; UKF, unscented Kalman filter.

**Table 4:** Dataset description.

Dataset description	
Training	3101
Testing	886
Validation	443
Total number of data	4430

**Table 5:** Computational time and computational complexity.

Filters	Computational time (s)
UKF	0.00184
EKF	0.01813
CDKF	0.02052

Abbreviations: CDKF, central difference Kalman filter; EKF, extended Kalman filter; UKF, unscented Kalman filter.

**Table 6:** Dataset generated from 2D Cartesian coordinates using various sensors and RSUs.

x_true	z_true	Velocity	x_RADAR	y_RADAR	x_LiDAR	y_LiDAR	x_RSU1	y_RSU1	x_RSU2	y_RSU2
−0.111607	−0.96288	3.814981	−0.156984	−0.940856	−0.054714	−0.948729	−0.0922501	−1.106488	−0.136862	−0.988507
−0.149753	−1.043139	3.7964	−0.145162	−1.003277	−0.111874	−1.02617	−0.189233	−0.975541	−0.030463	−1.272861
−0.340151	−1.037359	4.498759	−0.348351	−1.110464	−0.323851	−1.110081	−0.4950879	−1.050265	−0.377003	−1.042128
−0.638038	−0.888103	5.478234	−0.595089	−0.879368	−0.637221	−0.926621	−0.5071911	−0.949163	−0.703995	−0.869668
−0.593149	−0.905942	3.630605	−0.586281	−0.990257	−0.591301	−0.90488	−0.4308772	−0.715248	−0.755802	−0.689413
−0.673106	−0.819535	5.604542	−0.646965	−0.782227	−0.613923	−0.776927	−0.6329306	−0.713896	−0.787888	−0.8453
−0.637711	−0.990948	4.663502	−0.578478	−0.965164	−0.649438	−0.995931	−0.5356508	−1.027887	−0.554898	−1.023323
−0.570663	−1.117529	4.838129	−0.523111	−1.135418	−0.529771	−1.138704	−0.4440307	−1.006365	−0.660527	−1.069241
−0.67158	−1.038404	6.583375	−0.696376	−1.023598	−0.621968	−1.047042	−0.5207227	−0.907568	−0.724674	−0.927991
−0.764464	−0.9224	5.676109	−0.794376	−0.933887	−0.767135	−0.889105	−0.6853175	−0.720129	−0.698566	−0.919095
−0.846214	−0.907519	3.837177	−0.959468	−0.927619	−0.954005	−0.867395	−0.9759611	−1.073825	−0.928354	−0.95729
−0.85883	−0.933746	4.638115	−0.86312	−0.967483	−0.858406	−0.973409	−0.8573418	−0.888553	−0.784406	−1.01251
−0.881199	−0.867851	7.62291	−0.812112	−0.847388	−0.919095	−0.816449	−0.7871422	−0.731765	−0.894459	−0.685827
−0.851418	−0.849771	5.882437	−0.843759	−0.835746	−0.831645	−0.790567	−0.8853511	−0.832675	−0.898833	−1.018731
−0.731486	−0.832786	6.047708	−0.702867	−0.849719	−0.74364	−0.906319	−0.6536659	−0.718753	−0.750806	−0.988312
−0.88528	−0.948664	6.246795	−0.981415	−0.948936	−0.862056	−0.911324	−0.8761673	−0.905641	−0.97485	−0.911813
−0.981924	−0.753061	4.040738	−1.027307	−0.762205	−0.946865	−0.806897	−1.0391872	−0.697291	−1.130053	−0.899895
−1.010275	−0.695867	4.598584	−1.034805	−0.767027	−1.085875	−0.574401	−0.966065	−0.713223	−0.974945	−0.651302

*Note:* The sample dataset was generated from 2D Cartesian coordinates using various sensors and RSUs and some of the data from the dataset is presented in this table. It displays the position and velocity of nearby vehicles relative to the ego vehicle (i.e., the host vehicle), with *x*–*y* coordinates ranging from −1 to 1 on both the *x*-axis and *y*-axis, which correspond to longitude and latitude in the real world.

## Data Availability

The data will be made available upon request from the corresponding author.
